# Matched Filtering for MIMO Coherent Optical Communications with Mode-Dependent Loss Channels

**DOI:** 10.3390/s22030798

**Published:** 2022-01-21

**Authors:** Luis M. Torres, Francisco J. Cañete, Luis Díez

**Affiliations:** 1 Research and Development Department, KDPOF SL, 28760 Madrid, Spain; 2 Communications and Signal Processing Lab, Instituto Universitario de Investigación en Telecomunicación (TELMA), ETSI Telecomunicación Universidad de Málaga, 29071 Málaga, Spain; francis@ic.uma.es (F.J.C.); diez@ic.uma.es (L.D.)

**Keywords:** coherent optical communication, optical fiber communication, MIMO adaptive equalizer, matched filter, MMSE, spatial division multiplexing (SDM), polarization division multiplexing, fractional-spaced equalizer (FSE)

## Abstract

The use of digital signal processors (DSP) to equalize coherent optical communication systems based on spatial division multiplexing (SDM) techniques is widespread in current optical receivers. However, most of DSP implementation approaches found in the literature assume a negligible mode-dependent loss (MDL). This paper is focused on the linear multiple-input multiple-output (MIMO) receiver designed to optimize the minimum mean square error (MMSE) for a coherent SDM optical communication system, without previous assumptions on receiver oversampling or analog front-end realizations. The influence of the roll-off factor of a generic pulse-amplitude modulation (PAM) transmitter on system performance is studied as well. As a main result of the proposed approach, the ability of a simple match filter (MF) based MIMO receiver to completely eliminate inter-symbol interference (ISI) and crosstalk for SDM systems under the assumption of negligible MDL is demonstrated. The performance of the linear MIMO fractionally-spaced equalizer (FSE) receiver for an SDM system with a MDL-impaired channel is then evaluated by numerical simulations using novel system performance indicators, in the form of signal to noise and distortion ratio (SNDR) loss, with respect to the case without MDL. System performance improvements by increasing the transmitter roll-off factor are also quantified.

## 1. Introduction

The increasing demand of higher bit rates, combined with the environmental requirement of energy-efficient communication systems, is driving the development of ultra-high-capacity fiber optic communications. In this context, recent advances in spatial division multiplexing (SDM) using multimode or multicore fibers in long- and short-distance links [1,2] cannot be possible without the extensive use of multiple-input multiple-output (MIMO) signal processing.

Since the initial proposal to use polarization-division multiplexing (PDM) in a single mode fiber (SMF) [3] to double the capacity of a coherent optical communication system, MIMO signal processing [4,5] has become necessary to process and recover the parallel transmitted data streams even before the signal processing used was identified as a MIMO equalizer [6]. The channel model for PDM in SMF and its relation with the non-linear Schrödinger equation [7], its representation by means of the 2×2 Jones matrix [8] and as a multi-section system [9] has been extensively discussed in previous works. Multiple contributions to adaptive MIMO equalizers using the flexibility of digital signal processors (DSP) have been developed [7,10,11], where normally the equalization is divided into two parts: The first one, with an invariant chromatic dispersion (CD) compensation for each of the polarizations; and a second one, with an adaptive 2×2 MIMO linear equalizer to resolve the crosstalk between the modes [12].

SDM [13] using multimode fibers (MMF) [14] or few mode fibers (FMF) [15,16], appeared as a solution for communication systems reaching speeds well above 100 Tb/s when combined with wavelength multiplexing techniques [17]. Therefore, the optical channel model, based on the Jones matrix, was extended to represent the multiple fiber modes [18,19,20], and adaptive linear MIMO equalization [21] was studied and updated as an extension of the PDM case [2]. There are works that study the complexity of direct time and frequency domain implementations of the adaptive MIMO equalization, both for a linear design [12,22,23,24] and a nonlinear one [25], and also in the optical domain [26]. An important difference of SDM systems w.r.t. PDM systems is that the modal dispersion (MD) in SDM systems is higher than the equivalent polarization dispersion in PDM systems, reaching the same order of magnitude of the CD [20]. This boosts looking for simpler DSP schemes that avoid the enormous complexity required from the classical equalizers proposed for a PDM system and initially adapted to SDM systems [24]. In particular, linear MIMO receiver designs have been proposed for SDM systems [27,28] by expanding PDM systems [12,21,29], where a fractional-spaced equalizer (FSE) with an oversampling rate $r_{ov}$ of two is used. A review of different combinations of fiber types and DSP schemes reported in the literature with their associated complexity is summarized in [1].

The impact of mode-dependent loss (MDL) in long-haul optical links has more recently been studied, especially in the associated loss in the channel capacity when using minimum mean square error (MMSE) MIMO receivers [27,30]. This fact has initiated a race towards nonlinear receivers that can improve performance in the presence of MDL, increasing the receiver complexity notably [31,32]. However, performance evaluation of SDM systems that incorporate MIMO FSE receivers in the presence of MDL and the impact that pulse-amplitude modulation (PAM) pulses roll-off factor have on this performance, still deserve attention.

This paper provides a framework for the analysis of linear MIMO receivers for SDM that includes a continuous-time MIMO matched filter followed by a MIMO linear filter, without making prior assumptions about oversampling or the continuous-time optical front-end. This approach provides, for example, a theoretical basis for possible silicon photonics optical front-ends capable of SDM equalization. We show that the generalized linear MIMO MMSE receiver, for channels with negligible MDL, can be simplified to a matched filter MIMO receiver, which completely eliminates the ISI and crosstalk introduced by the channel.

When the optical channel exhibits a significant MDL, we include linear equalization and carry out numerical simulations to get the performance of a system that consists of: A PAM transmitter with square-root raised cosine pulses; a complete long-haul optical channel with SDM; and a MIMO receiver based on the FSE approach with oversampling of two. To this end, an ensemble of thousands of random optical channels has been generated and the system performance is evaluated by means of the signal to noise and distortion ratio (SNDR) loss at the receiver output w.r.t., the one of an optimal equivalent system without ISI and crosstalk. These results are presented for a configuration with a set of parameters for a fiber, transmitter, and receiver, which is representative of current technology.

The paper is structured as follows. After a short section of defining the notation used (Section 2), we begin by describing the optical channel model for a long-haul communication system using SDM, including CD, MD, and MDL impairments (Section 3). Next, a communication system based on a generalized PAM transmitter with square-root raised cosine pulses, and a linear MIMO receiver designed under the MMSE optimization criterion are discussed in Section 4. In Section 5, the numerical simulations are presented and SNDR loss metrics are given for the optical communication system with a FSE MIMO receiver for different values of channel dispersion (including MDL) and roll-off factor of the square-root raised cosine pulses. Finally, conclusions are summarized in Section 6.

## 2. Notation

Matrices are represented as $\underline{\underline{M}}$, and vectors as $\underline{v}$. Vectors are column vectors unless otherwise noted. ⌊x⌋ denotes the largest integer less than or equal to *x*. $x^{*}$ represents the conjugate of *x*, ${\underline{\underline{M}}}^{H}$ denotes the Hermitian of $\underline{\underline{M}}$, and ${\underline{v}}^{T}$ represents the transpose of the vector $\underline{v}$. E[x] is the expectation operator applied to the random variable *x*. i∈{1,…,D} is used to index a mode among the *D* modes used in the fiber and ∗ represents the convolution operator. The result of the convolution operator applied to a $D_{1}\times D_{2}$ matrix $\underline{\underline{a}}\left(t\right)$ and a $D_{2}\times D_{3}$ matrix $\underline{\underline{b}}\left(t\right)$ is a $D_{1}\times D_{3}$ matrix denoted as $\underline{\underline{c}}\left(t\right)$ and given by:

(1) $$\underline{\underline{c}}\left(t\right)=\underline{\underline{a}}\left(t\right)*\underline{\underline{b}}\left(t\right)$$

where each of the elements of $\underline{\underline{c}}\left(t\right)$, denoted as $c_{ij}\left(t\right)$, are obtained as in a simple matrix multiplication, but substituting the product by the convolution operator:

(2) $$c_{ij}\left(t\right)=\sum_{k=1}^{D_{2}}a_{ik}\left(t\right)*b_{kj}\left(t\right).$$

Similarly, the result of the convolution operator applied to a $D_{1}\times D_{2}$ matrix $\underline{\underline{a}}\left(t\right)$ and a time dependent signal y(t) is a $D_{1}\times D_{2}$ matrix denoted as $\underline{\underline{d}}\left(t\right)$ where $\underline{\underline{d}}\left(t\right)=\underline{\underline{a}}\left(t\right)*y\left(t\right)$. Each of the elements of the matrix $\underline{\underline{d}}\left(t\right)$ are obtained as in a multiplication of a matrix with a scalar, however substituting the product by the convolution operator:

(3) $$d_{ij}\left(t\right)=a_{ij}\left(t\right)*y\left(t\right).$$

F{y(t)} denotes the Fourier transform of the continuous-time signal y(t) and $F^{-1}\left\{Y\left(\omega \right)\right)\}$ the denotes inverse Fourier transform of Y(ω). Similarly, for the discrete-time signal y[n] we denote its corresponding discrete Fourier transform as Y(Ω).

## 3. Long-Haul Optical Link MIMO Channel Model

In this section we describe the multi-section optical channel model used in this work. The effect of the channel noise is discussed separately in Section 4. The relationship between the input vector $\underline{x}\left(\omega \right)={\left[x_{1}\left(\omega \right),x_{2}\left(\omega \right),\ldots ,x_{D}\left(\omega \right)\right]}^{T}$ of complex electric field amplitudes of each of the *D* modes propagating along the fiber, and the corresponding output vector $\underline{y}\left(\omega \right)={\left[y_{1}\left(\omega \right),y_{2}\left(\omega \right),\ldots ,y_{D}\left(\omega \right)\right]}^{T}$ can be modeled, after neglecting non-linear effects, as a multiple-input multiple-output linear system ${\underline{\underline{H}}}_{tot}\left(\omega \right)$ [2]:

(4) $$\underline{y}\left(\omega \right)={\underline{\underline{H}}}_{tot}\left(\omega \right)\underline{x}\left(\omega \right),$$

where ${\underline{\underline{H}}}_{tot}\left(\omega \right)$ is a D×D matrix that models the signal propagation along the channel. For D=2, the system is equivalent to a classical PDM over a SMF, and ${\underline{\underline{H}}}_{tot}\left(\omega \right)$ takes the form of the Jones matrix [8]. For D>2, extensions to the Jones matrix have been proposed to be adequate for the SDM model [19,20].

In the case of long-haul systems, ${\underline{\underline{H}}}_{tot}\left(\omega \right)$ can be further modeled as a concatenation of $K_{amp}$ spans composed of the optical fiber and an optical amplifier [2,18,33,34]. Hence, the whole channel transfer function can be written as:

(5) $${\underline{\underline{H}}}_{tot}\left(\omega \right)=H_{CD}\left(\omega \right)\cdot \underline{\underline{H}}\left(\omega \right),$$

where $H_{CD}\left(\omega \right)=e^{\left(-\frac{j}{2}\omega^{2}{\bar{\beta }}_{2}\ell_{tot}\right)}$ is a single-input single-output (SISO) term that models the mode-averaged distortion due to CD, ${\bar{\beta }}_{2}$ represents the mode-averaged CD per unit length, and $\ell_{tot}$ denotes the total link length. The matrix $\underline{\underline{H}}\left(\omega \right)$ includes inter-mode cross-talk, MDL and MD effects of the complete MIMO system. Equation (5) can be written as a product over the $K_{amp}$ spans:

(6) $$\underline{\underline{H}}\left(\omega \right)=\prod_{k=1}^{K_{amp}}{\underline{\underline{H}}}^{\left(k\right)}\left(\omega \right),$$

where ${\underline{\underline{H}}}^{\left(k\right)}\left(\omega \right)$ is the channel response of the *k*th span. We use $k\in \{1,\ldots ,K_{amp}\}$ to index the spans in the optical channel. We can write out ${\underline{\underline{H}}}^{\left(k\right)}\left(\omega \right)$ as [20,34]:

(7) $${\underline{\underline{H}}}^{\left(k\right)}\left(\omega \right)={\underline{\underline{V}}}^{\left(k\right)}{\underline{\underline{\Lambda }}}^{\left(k\right)}\left(\omega \right){\left({\underline{\underline{U}}}^{\left(k\right)}\right)}^{H},$$

where the diagonal matrix ${\underline{\underline{\Lambda }}}^{\left(k\right)}\left(\omega \right)$ for a given span *k* includes the MDL effects and the MD of each mode w.r.t. the mode-averaged value [33], and can be expressed as:

(8) $${\underline{\underline{\Lambda }}}^{\left(k\right)}\left(\omega \right)=\mathrm{diag}\left(\left[e^{\left(\frac{1}{2}g_{1}^{\left(k\right)}-j\omega \tau_{1}^{\left(k\right)}\right)},\ldots ,e^{\left(\frac{1}{2}g_{D}^{\left(k\right)}-j\omega \tau_{D}^{\left(k\right)}\right)}\right]\right),$$

being ${\underline{g}}^{\left(k\right)}=\left[g_{1}^{\left(k\right)},g_{2}^{\left(k\right)},\ldots ,g_{D}^{\left(k\right)}\right]$ the uncoupled modal gains and ${\underline{\tau }}^{\left(k\right)}=\left[\tau_{1}^{\left(k\right)},\tau_{2}^{\left(k\right)},\ldots ,\tau_{D}^{\left(k\right)}\right]$ uncoupled modal group delays. We assume that the uncoupled modal group-velocity dispersion is equal to zero for all the *k* spans [33].

The *k*-th span mode coupling is modeled by the frequency-independent ${\underline{\underline{V}}}^{\left(k\right)}$ and ${\underline{\underline{U}}}^{\left(k\right)}$ matrices. It is important to note that, by considering that all the modes propagating through the fiber experience the same attenuation, both matrices are unitary, i.e.,

(9) $${\underline{\underline{V}}}^{\left(k\right)}\cdot {\left({\underline{\underline{V}}}^{\left(k\right)}\right)}^{H}=\underline{\underline{I}}={\underline{\underline{U}}}^{\left(k\right)}\cdot {\left({\underline{\underline{U}}}^{\left(k\right)}\right)}^{H}.$$

Alternatively, $\underline{\underline{H}}\left(\omega \right)$ can also be written by applying a singular value decomposition (SVD), as the product of two unitary matrices ${\underline{\underline{U}}}^{\left(tot\right)}\left(\omega \right)$ and ${\underline{\underline{V}}}^{\left(tot\right)}\left(\omega \right)$, and a diagonal matrix ${\underline{\underline{\Lambda }}}^{\left(tot\right)}\left(\omega \right)$ as [35]:

(10) $$\underline{\underline{H}}\left(\omega \right)={\underline{\underline{V}}}^{\left(tot\right)}\left(\omega \right){\underline{\underline{\Lambda }}}^{\left(tot\right)}\left(\omega \right){\underline{\underline{U}}}^{{\left(tot\right)}^{H}}\left(\omega \right)$$

where now, the diagonal matrix ${\underline{\underline{\Lambda }}}^{\left(tot\right)}\left(\omega \right)$ is given by:

(11) $${\underline{\underline{\Lambda }}}^{\left(tot\right)}\left(\omega \right)=\mathrm{diag}\left(\left[e^{\left(\frac{1}{2}g_{1}^{\left(tot\right)}-j\omega \tau_{1}^{\left(tot\right)}\right)},\ldots ,e^{\left(\frac{1}{2}g_{D}^{\left(tot\right)}-j\omega \tau_{D}^{\left(tot\right)}\right)}\right]\right)$$

where ${\underline{g}}^{\left(tot\right)}=\left[g_{1}^{\left(tot\right)},g_{2}^{\left(tot\right)},\ldots ,g_{D}^{\left(tot\right)}\right]$ are the coupled modal gains of the overall channel and ${\underline{\tau }}^{\left(tot\right)}=\left[\tau_{1}^{\left(tot\right)},\tau_{2}^{\left(tot\right)},\ldots ,\tau_{D}^{\left(tot\right)}\right]$ denote the coupled modal group delays.

Note that in (10), both ${\underline{\underline{U}}}^{\left(tot\right)}\left(\omega \right)$ and ${\underline{\underline{V}}}^{\left(tot\right)}\left(\omega \right)$ unitary matrices have in general frequency dependence, in contrast to ${\underline{\underline{U}}}^{\left(k\right)}$ and ${\underline{\underline{V}}}^{\left(k\right)}$ in (7) that have not [27,33].

## 4. SDM Communication System Model

**Figure 1:**
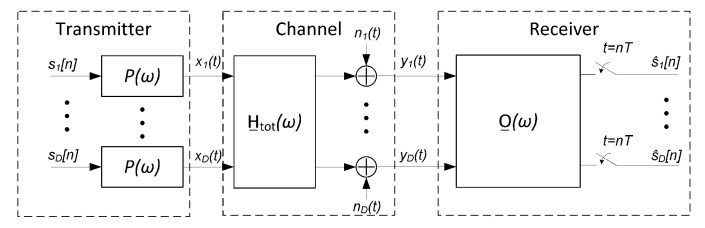
Spatial division multilpexing (SDM) communication system model with linear multiple-input multiple-output (MIMO) receiver.

This section describes the model employed to represent the communication system established over the optical channel with multiple spans. The SVD of the channel in (10) can be useful for designing a transmitter based on a precoding matrix combined with a linear receiver, as used in wireless systems [36]. However, this approach becomes unfeasible for long-haul optical communication systems, since the end-to-end channel side information needed to build the transmitter precoding matrix changes faster than the time needed for the system to collect, send, and process that information [33]. Therefore we focus on a SDM system with no channel side information that uses a linear receiver to cope with the channel impairments as shown in Figure 1 [37].

The binary data symbols, $\underline{s}\left[n\right]={\left[s_{1}\left[n\right],s_{2}\left[n\right],\ldots ,s_{D}\left[n\right]\right]}^{T}$, are PAM modulated in parallel for each of the i∈{1,…,D} optical modes using the same transmitter pulse P(ω) to get the PAM signals, denoted by the column vector $\underline{x}\left(t\right)={\left[x_{1}\left(t\right),x_{2}\left(t\right),\ldots ,x_{D}\left(t\right)\right]}^{T}$. In Figure 1, *T* is the transmitted symbol period and the first block represent *D* parallel PAM modulators working at a symbol rate (and, hence, it includes the discrete-time to continuous-time conversion). The transmitted signal is distorted by ISI and crosstalk introduced by the MIMO channel, modeled with the ${\underline{\underline{H}}}_{tot}\left(\omega \right)$ matrix described in Section 3. It has been shown that the noise in a MDL-impaired system is additive and spatially white [21]. Therefore, in this work we add before the receiver, as part of the channel, an additive white Gaussian noise (AWGN) vector $\underline{n}\left(t\right)={\left[n_{1}\left(t\right),n_{2}\left(t\right),\ldots ,n_{D}\left(t\right)\right]}^{T}$, which, for a certain mode *i*, has a variance equal to $\frac{N_{0}}{2}$.

The resulting continuous-time signal vector $\underline{y}\left(t\right)={\left[y_{1}\left(t\right),y_{2}\left(t\right),\ldots ,y_{D}\left(t\right)\right]}^{T}$ is processed by a MIMO receiver to obtain the estimation of the transmitted symbols $\underline{s}\left[n\right]$, denoted as $\underline{\hat{s}}\left[n\right]={\left[{\hat{s}}_{1}\left[n\right],{\hat{s}}_{2}\left[n\right],\ldots ,{\hat{s}}_{D}\left[n\right]\right]}^{T}$. In this work we focus on linear MIMO receivers and so, the estimation part in the receiver is depicted in Figure 1 with a generic linear filter of response $\underline{\underline{O}}\left(\omega \right)$, which is followed by a sampler working at the symbol rate. In the following, we propose linear MIMO receiver structures based on the MMSE criterion of an estimated symbol vector.

### 4.1. Transmitter

The transmitted data in each of the *D* modes are modulated using a PAM with a square-root raised cosine pulse p(t) with roll-off factor equal to α and normalized power, which can be expressed as [38]:

(12) $$p\left(t\right)=\frac{4\alpha }{\pi \sqrt{T}}\cdot \frac{\cos\left(\left[1+\alpha \right]\frac{\pi t}{T}\right)+\frac{T\cdot \sin\left(\left[1-\alpha \right]\frac{\pi t}{T}\right)}{4\alpha t}}{1-{\left(\frac{4\alpha t}{T}\right)}^{2}}.$$

Hence, we can write the sequence of PAM pulses for a given mode *i* as:

(13) $$x_{i}\left(t\right)=\sum_{n=-\infty }^{\infty }s_{i}\left[n\right]p\left(t-nT\right),$$

where $s_{i}\left[n\right]$ is a random variable with values taken from the set defined by the PAM modulation scheme. Let us define the global impulse response $\underline{\underline{q}}\left(t\right)$ as the convolution of the transmitting pulse p(t) and the optical channel impulse response matrix ${\underline{\underline{h}}}_{tot}\left(t\right)$ as:

(14) $$\underline{\underline{q}}\left(t\right)={\underline{\underline{h}}}_{tot}\left(t\right)*p\left(t\right),$$

so that $\underline{\underline{q}}\left(t\right)$ is a D×D matrix of impulse responses. This way, $q_{ij}\left(t\right)$ describes the impulse response between the transmitter mode *i* and the receiver mode *j*. Therefore, we can write the relationship between the transmitted symbols $s_{j}\left[n\right]$ and the received signal in mode *i*, $y_{i}\left(t\right)$, as:

(15) $$y_{i}\left(t\right)=\sum_{n}\sum_{j=1}^{D}s_{j}\left[n\right]q_{ij}\left(t-nT\right)+n_{i}\left(t\right)$$

with $n_{i}\left(t\right)$ as the noise in the *i*-th receiver mode.

### 4.2. Linear MMSE MIMO Receiver

The most widely used linear MIMO receiver for SDM systems is based on the design of filter $\underline{\underline{O}}\left(\omega \right)$ in Figure 1 to minimize the mean squared error (MSE), which is called a linear MMSE MIMO receiver [28,37,39]. Mathematically, the MSE for the linear MIMO receiver under the MMSE criterion is defined as:

(16) $$\sigma_{\mathrm{MMSE}-\mathrm{LE}}^{2}=E\left[{\underline{e}}^{H}\left[n\right]\underline{e}\left[n\right]\right]$$

where

(17) $$\underline{e}\left[n\right]=\underline{s}\left[n\right]-\underline{\hat{s}}\left[n\right]$$

with $\underline{\hat{s}}\left[n\right]$ as the output vector of the linear MIMO receiver $\underline{\underline{O}}\left(\omega \right)$.

**Figure 2:**
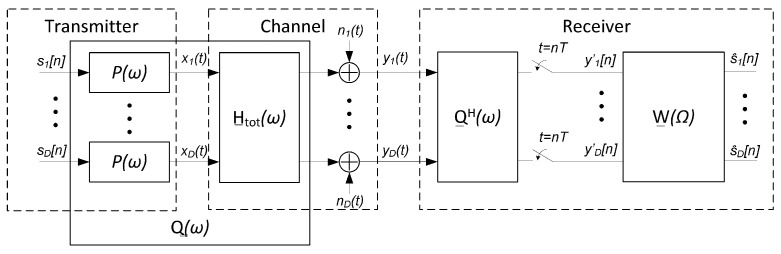
SDM communication system model with linear minimum mean square error (MMSE) MIMO receiver.

It is well known that the structure of a linear MMSE MIMO receiver can be divided into a matched filter ${\underline{\underline{Q}}}^{H}\left(\omega \right)$ operating in continuous time, a sampler operating at the symbol rate, and a discrete-time equalizer of response $\underline{\underline{W}}\left(\Omega \right)$, as presented in Figure 2.

We denote as $\underline{y^{\prime }}\left[n\right]$ the vector of samples after the match filter ${\underline{\underline{Q}}}^{H}\left(\omega \right)=F\left\{{\underline{\underline{q}}}^{H}\left(-t\right)\right\}$ in the receiver and a symbol rate sampling, being $\underline{\underline{q}}\left(t\right)=F^{-1}\left\{\underline{\underline{Q}}\left(\omega \right)\right\}$ defined in (14). Now, we define the sampled impulse response at t=nT of the convolution of $\underline{\underline{q}}\left(t\right)$ and its matched filter ${\underline{\underline{q}}}^{H}\left(-t\right)$, which represents the equivalent discrete channel response as:

(18) $$\underline{\underline{g}}\left[n\right]={\left.\underline{\underline{q}}\left(t\right)*{\underline{\underline{q}}}^{H}\left(-t\right)\right|}_{t=nT},$$

and its discrete Fourier transform pair as:

(19) $$\underline{\underline{G}}\left(\Omega \right)=\sum_{n=-\infty }^{\infty }\underline{\underline{g}}\left[n\right]\cdot e^{-j\Omega n}.$$

Hence, the optimal discrete-time MIMO equalizer ${\underline{\underline{W}}}_{opt}\left(\Omega \right)$ according to the MMSE criterion becomes:

(20) $$\begin{array}{c} {\underline{\underline{W}}}_{opt}\left(\Omega \right)={\left[\underline{\underline{G}}\left(\Omega \right)+\underline{\underline{I}}\cdot \left(\frac{N_{0}}{2}\right)\right]}^{-1}, \end{array}$$

where we are considering a normalized transmission power equally distributed in each of the *D* modes.

When $\underline{\underline{G}}\left(\Omega \right)$ satisfies the Nyquist criterion for MIMO systems $\underline{\underline{G}}\left(\Omega \right)=\underline{\underline{I}}$, there is neither ISI nor cross-talk at the matched filter output, further equalization would not be needed, and the optimum linear receiver consists only in the matched filter. However, if such a criterion is not fulfilled, the equalizer $\underline{\underline{W}}\left(\Omega \right)$ is essential and some SNDR loss at the output will be unavoidable w.r.t. the ideal case.

### 4.3. Matched Filter-Based Receiver for SDM

In this subsection we will explore the optical channel requirements to reduce the linear MIMO receiver $\underline{\underline{O}}\left(\omega \right)$ in Figure 1 to a simple matched filter-based receiver. Furthermore, we will show that the resulting receiver is optimal in the sense that the discrete-time system response of the SDM communication system is the identity matrix, followed by the addition of the AWGN noise.

Let us first write out:

(21) $$\underline{\underline{Q}}\left(\omega \right)=P\left(\omega \right)\cdot {\underline{\underline{H}}}_{tot}\left(\omega \right),$$

where ${\underline{\underline{H}}}_{tot}\left(\omega \right)$ and P(ω) are the Fourier transforms of ${\underline{\underline{h}}}_{tot}\left(t\right)$ and p(t), respectively and according to what is plotted in Figure 2. It follows that:

(22) $$\underline{\underline{Q}}{\left(\omega \right)}^{H}=P^{*}\left(\omega \right)\cdot {\underline{\underline{H}}}_{tot}{\left(\omega \right)}^{H}.$$

**Figure 3:**
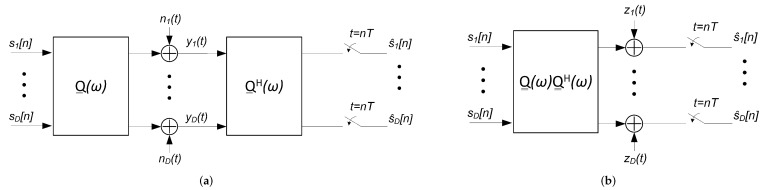
SDM communication system model with matched filter-based receiver (**a**) and its reordered version (**b**).

The signal at each of the *D* branches $y_{i}\left(t\right)$, defined in (15), is processed before sampling by the continuous-time filter ${\underline{\underline{Q}}}^{H}\left(\omega \right)$. The equivalent scheme for this matched filter-based MIMO receiver is shown in Figure 3a. By using the linearity of the system we can rearrange Figure 3a to obtain Figure 3b. Then, elaborating the expression $\underline{\underline{Q}}\left(\omega \right){\underline{\underline{Q}}}^{H}\left(\omega \right)$ we obtain that:

(23) $$\underline{\underline{Q}}\left(\omega \right){\underline{\underline{Q}}}^{H}\left(\omega \right)=P\left(\omega \right)\cdot {\underline{\underline{H}}}_{tot}\left(\omega \right)\cdot {\underline{\underline{H}}}_{tot}^{H}\left(\omega \right)\cdot P^{*}\left(\omega \right)=P\left(\omega \right)\cdot \underline{\underline{H}}\left(\omega \right)\cdot {\underline{\underline{H}}}^{H}\left(\omega \right)\cdot P^{*}\left(\omega \right),$$

where we have used that:

(24) $$H_{CD}\left(\omega \right)\cdot H_{CD}^{*}\left(\omega \right)=1.$$

From (10) we have that:

(25) $$\begin{array}{cc} \underline{\underline{H}}\left(\omega \right)\cdot \underline{\underline{H}}{\left(\omega \right)}^{H} & =\\ \; & \left(\prod_{k=1}^{K_{amp}-1}{\underline{\underline{V}}}^{\left(k\right)}{\underline{\underline{\Lambda }}}^{\left(k\right)}\left(\omega \right){\underline{\underline{U}}}^{{\left(k\right)}^{H}}\right)\cdot {\underline{\underline{V}}}^{\left(K_{amp}\right)}{\underline{\underline{\Lambda }}}^{\left(K_{amp}\right)}\left(\omega \right){{\underline{\underline{U}}}^{\left(K_{amp}\right)}}^{H}\\ \; & \cdot {\underline{\underline{U}}}^{\left(K_{amp}\right)}{\underline{\underline{\Lambda }}}^{{\left(K_{amp}\right)}^{H}}\left(\omega \right){\underline{\underline{V}}}^{{\left(K_{amp}\right)}^{H}}\\ \; & \cdot \left(\prod_{k=2}^{K_{amp}}{\underline{\underline{U}}}^{\left(K_{amp}-k+1\right)}{\underline{\underline{\Lambda }}}^{{\left(K_{amp}-k+1\right)}^{H}}\left(\omega \right){\underline{\underline{V}}}^{{\left(K_{amp}-k+1\right)}^{H}}\right)\\ \; & =\left(\prod_{k=1}^{K_{amp}-1}{\underline{\underline{V}}}^{\left(k\right)}{\underline{\underline{\Lambda }}}^{\left(k\right)}\left(\omega \right){\underline{\underline{U}}}^{{\left(k\right)}^{H}}\right)\cdot {\underline{\underline{V}}}^{\left(K_{amp}\right)}\cdot {\left|{\underline{\underline{\Lambda }}}^{\left(K_{amp}\right)}\left(\omega \right)\right|}^{2}\cdot {\underline{\underline{V}}}^{{\left(K_{amp}\right)}^{H}}\\ \; & \cdot \left(\prod_{k=2}^{K_{amp}}{\underline{\underline{U}}}^{\left(K_{amp}-k+1\right)}{\underline{\underline{\Lambda }}}^{{\left(K_{amp}-k+1\right)}^{H}}\left(\omega \right){\underline{\underline{V}}}^{{\left(K_{amp}-k+1\right)}^{H}}\right). \end{array}$$

And the diagonal matrix:

(26) $$\begin{array}{ccc} |{\underline{\underline{\Lambda }}}^{\left(K_{amp}\right)}{\left(\omega \right)|}^{2} & =\\ & \mathrm{diag}\left(\left[e^{\frac{1}{2}g_{1}^{\left(K_{amp}\right)}-j\omega \tau_{1}^{\left(K_{amp}\right)}},\ldots ,e^{\frac{1}{2}g_{D}^{\left(K_{amp}\right)}-j\omega \tau_{D}^{\left(K_{amp}\right)}}\right]\right)\cdot \\ \; & \mathrm{diag}\left(\left[e^{\frac{1}{2}g_{1}^{\left(K_{amp}\right)}+j\omega \tau_{1}^{\left(K_{amp}\right)}},\ldots ,e^{\frac{1}{2}g_{D}^{\left(K_{amp}\right)}+j\omega \tau_{D}^{\left(K_{amp}\right)}}\right]\right) & \\ \; & ={\left|\mathrm{diag}\left(\left[e^{\frac{1}{2}g_{1}^{\left(K_{amp}\right)}},\ldots ,e^{\frac{1}{2}g_{D}^{\left(K_{amp}\right)}}\right]\right)\right|}^{2} \end{array}$$

that does not allow simplifying (25) unless the following holds:

(27) $$|{\underline{\underline{\Lambda }}}^{\left(K_{amp}\right)}{\left(\omega \right)|}^{2}=e^{\left(g^{\left(K_{amp}\right)}\right)}\cdot \underline{\underline{I}}.$$

This latter condition is equivalent to assuming that:

(28) $$e^{\left(\frac{1}{2}g^{\left(K_{amp}\right)}\right)}=e^{\left(\frac{1}{2}g_{1}^{\left(K_{amp}\right)}\right)}=e^{\left(\frac{1}{2}g_{2}^{\left(K_{amp}\right)}\right)}=\cdots =e^{\left(\frac{1}{2}g_{D}^{\left(K_{amp}\right)}\right)},$$

or, in other words, that the MDL is negligible for the $K_{amp}$-th span. When the condition expressed in (27) is satisfied for all the spans of the system, we can commute the terms in (25), and therefore, we can obtain:

(29) $$\underline{\underline{H}}\left(\omega \right)\cdot \underline{\underline{H}}{\left(\omega \right)}^{H}=\prod_{k=1}^{K_{amp}}|{\underline{\underline{\Lambda }}}^{\left(k\right)}{\left(\omega \right)|}^{2}=\prod_{k=1}^{K_{amp}}e^{\left(g^{\left(k\right)}\right)}\cdot \underline{\underline{I}}=e^{\left(\sum_{k=1}^{K_{amp}}g^{\left(k\right)}\right)}\cdot \underline{\underline{I}}\;.$$

Revisiting (23), and plugging in (29) under the assumption of a negligible MDL in the optical channel, we can write:

(30) $$\underline{\underline{G}}\left(\omega \right)=\underline{\underline{Q}}\left(\omega \right)\cdot {\underline{\underline{Q}}}^{H}\left(\omega \right)={\left|P\left(\omega \right)\right|}^{2}\cdot e^{\left(\sum_{k=1}^{K_{amp}}g^{\left(k\right)}\right)}\cdot \underline{\underline{I}}\;.$$

Therefore, without loss of generality, $e^{\left(\sum_{k=1}^{K_{amp}}g^{\left(k\right)}\right)}=1$ can be assumed and, after sampling at the symbol rate, (30) can be written as:

(31) $$\underline{\underline{G}}\left(\Omega \right)=\frac{1}{T}\cdot \sum_{l=-\infty }^{\infty }{\left|P\left(\frac{\Omega +2\pi l}{T}\right)\right|}^{2}\cdot \underline{\underline{I}}=\underline{\underline{I}},$$

where we have used that p(t) defined in Equation (12) is a square-root raised cosine pulse satisfying the Nyquist criterion.

**Figure 4:**
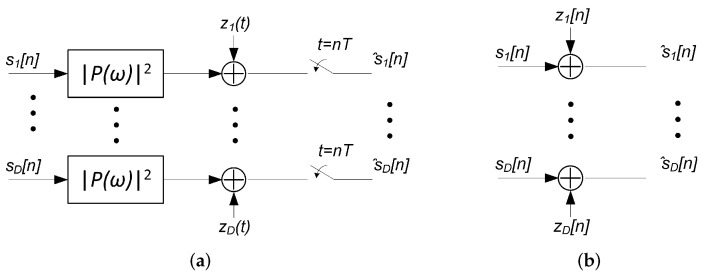
MIMO coherent optical communication system model with matched filter-based receiver in the absence of mode-dependent loss (MDL) (**a**) and its equivalent discrete-time system model (**b**).

Regarding the filtered noise waveforms $z_{1}\left(t\right)$ to $z_{D}\left(t\right)$ in Figure 4a, they have an autocorrelation function matrix ${\underline{\underline{R}}}_{zz}\left(t\right)$, whose Fourier transform pair ${\underline{\underline{S}}}_{z}\left(\omega \right)$ can be expressed as:

(32) $${\underline{\underline{S}}}_{z}\left(\omega \right)=\underline{\underline{Q}}\left(\omega \right)\cdot {\underline{\underline{S}}}_{n}\left(\omega \right)\cdot {\underline{\underline{Q}}}^{H}\left(\omega \right),$$

where ${\underline{\underline{S}}}_{n}\left(\omega \right)$ is the Fourier transform of the autocorrelation function matrix ${\underline{\underline{R}}}_{nn}\left(t\right)=\underline{\underline{I}}\cdot \frac{N_{0}}{2}\cdot \delta \left(t\right)$ of the received noise vector $\underline{n}\left(t\right)={\left[n_{1}\left(t\right),n_{2}\left(t\right),\ldots ,n_{D}\left(t\right)\right]}^{T}$. We remind that the noise components of the noise vector $\underline{n}\left(t\right)$ were assumed uncorrelated with identical power in each mode equal to $N_{0}/2$. Using (30) and (32) leads to:

(33) $${\underline{\underline{S}}}_{z}\left(\omega \right)=\frac{N_{0}}{2}\cdot \underline{\underline{Q}}\left(\omega \right)\cdot {\underline{\underline{Q}}}^{H}\left(\omega \right)=\frac{N_{0}}{2}\cdot {\left|P\left(\omega \right)\right|}^{2}\cdot e^{\left(\sum_{k=1}^{K_{amp}}g^{\left(k\right)}\right)}\cdot \underline{\underline{I}}.$$

Using the previous assumptions about gains $g^{\left(k\right)}$ and P(ω) made before, we obtain that the sampled noise vector $\underline{z}\left[n\right]={\left[z_{1}\left[n\right],\ldots ,z_{D}\left[n\right]\right]}^{T}$ has an autocorrelation matrix function ${\underline{\underline{R}}}_{zz}\left[n\right]=\underline{\underline{I}}\cdot \frac{N_{0}}{2}\cdot \delta \left[n\right]$.

Therefore, we can conclude that a D×D MIMO coherent optical communication system using a continuous-time matched filter as a receiver completely eliminates channel ISI and crosstalk when the MDL in the channel is negligible. Moreover, the equivalent discrete-time system model reduces to *D* discrete parallel AWGN channels as shown in Figure 4b. Hence, there would be no loss of performance w.r.t. the AWGN channel without distortion.

## 5. Numerical Simulation of Linear MIMO FSE Receiver for MDL-Impaired Optical Channel

**Figure 5:**
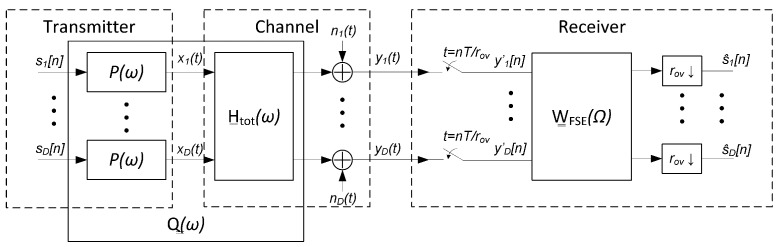
SDM communication system model with linear fractionally-spaced equalizer (FSE) MIMO receiver and integer oversampling rate $r_{ov}$.

In this section, we assess the performance of the ideal MMSE linear receiver when MDL is present. Specifically, we study the SNDR degradation at the receiver output w.r.t. the case when the MDL is negligible. To carry out this study, we will use a FSE-based receiver, as shown in Figure 5, which is the most common implementation of the ideal linear filter in discrete-time systems (see $\underline{\underline{O}}\left(\omega \right)$ in Figure 1). Note that this scheme is only valid for integer oversampling rates $r_{ov}$.

The FSE oversampling rate $r_{ov}$ has been set to two [1,12] and the discrete-time equalizer, with a ${\underline{\underline{W}}}_{FSE}\left(\Omega \right)$ response, has been designed with a number of taps $N_{taps}$ large enough so that any further increase does not lead to a significantly better SNDR at the receiver output. The decimated output of ${\underline{\underline{W}}}_{FSE}\left(\Omega \right)$, by a $r_{ov}$ factor, are the estimated symbol $\hat{\underline{s}}\left[n\right]$.

We define the receiver performance metric for each mode *i*, L(i), as the difference in dB between the output SNR of an ISI and crosstalk-free system with *D* parallel AWGN channels (see Figure 4b), and the output SNDR of the FSE-based receiver, denoted as ${\underline{SNDR}}_{out}$.

### 5.1. Channel Model

We decide to carry out the numerical simulations of the $\underline{\underline{H}}\left(\omega \right)$ channel model described in (6) in the time domain, so that the relative delays of the different modes can be easily described as a time shift between them. The different mode amplitudes can also be handled simply by a diagonal matrix. The chromatic dispersion, which have a SISO frequency response $H_{CD}\left(\omega \right)$ that does not depend on the mode i∈{0,…,D}, is represented as [7]:

(34) $$H_{CD}\left(\omega \right)=e^{-j\beta \frac{\omega^{2}}{2}},$$

where $\beta ={\bar{\beta }}_{2}\ell_{tot}$, and $\ell_{tot}=K_{amp}\ell_{span}$ when all spans are considered of equal length.

The MDL effect is modeled with an amplification factor for each mode and each optical amplifier (located at the end of each span). These factors are considered time-invariant for a given channel realization in the form of a vector for the *k*-th span ${\underline{g}}^{\left(k\right)}=\left[g_{1}^{\left(k\right)},g_{2}^{\left(k\right)},\ldots ,g_{D}^{\left(k\right)}\right]$, where $g_{i}^{\left(k\right)}$ for i∈{0,…,D} is expressed in dB and taken from a Gaussian distribution with zero mean and standard deviation (STD) $\sigma_{g}$. The sum of all factors $\sum_{i=1}^{D}g_{i}^{\left(k\right)}$ is set to 0 for normalization purposes. Hence, the amplitudes matrix of the *k*-th span, frequency independent, is given by:

(35) $$\begin{array}{c} {\underline{\underline{A}}}^{\left(k\right)}=\mathrm{diag}\left(\left[e^{\left(\frac{1}{2}g_{1}^{\left(k\right)}\right)},\ldots ,e^{\left(\frac{1}{2}g_{D}^{\left(k\right)}\right)}\right]\right). \end{array}$$

Alternatively, for each span *k* of the communication link we have the delays matrix:

(36) $$\begin{array}{c} {\underline{\underline{\Lambda }}}^{\left(k\right)}\left(\omega \right)={\underline{\underline{A}}}^{\left(k\right)}\cdot \mathrm{diag}\left(\left[e^{-j\omega \tau_{1}^{\left(k\right)}},\ldots ,e^{-j\omega \tau_{D}^{\left(k\right)}}\right]\right), \end{array}$$

being ${\underline{\tau }}^{\left(k\right)}=\left[\tau_{1}^{\left(k\right)},\tau_{2}^{\left(k\right)},\ldots ,\tau_{D}^{\left(k\right)}\right]$ the vector that models the MD with group delays for each mode of the *k*-th span.

To obtain the delays, we generate the first D/2 values of ${\underline{\tau }}^{\left(k\right)}$ from a Gaussian distribution with STD $\sigma_{gd}$, and the second D/2 values are taken as the opposite of these, which satisfies that $\sum_{i=1}^{D}\tau_{i}^{\left(k\right)}=0$, since we consider that the system uses polarization multiplexing as part of the SDM [40].

The time-domain impulse response for each of the spans *k* is calculated by applying the inverse Fourier transform to (7) and can be expressed as:

(37) $${\underline{\underline{h}}}^{\left(k\right)}\left(t\right)={\underline{\underline{V}}}^{\left(k\right)}{\underline{\underline{A}}}^{\left(k\right)}{\underline{\underline{d}}}^{\left(k\right)}\left(t\right){\underline{\underline{U}}}^{\left(k\right)},$$

where

(38) $${\underline{\underline{d}}}^{\left(k\right)}\left(t\right)=\mathrm{diag}\left(\left[\delta \left(t-\tau_{1}^{\left(k\right)}\right),\ldots ,\delta \left(t-\tau_{D}^{\left(k\right)}\right)\right]\right),$$

and we have used that the matrices ${\underline{\underline{A}}}^{\left(k\right)}$, ${\underline{\underline{U}}}^{\left(k\right)}$, and ${\underline{\underline{V}}}^{\left(k\right)}$ are constant.

Equation (37) describes that incoming signal at the *k*th span is multiplied by the unitary matrix ${\underline{\underline{U}}}^{\left(k\right)}$, then each modal impulse response is delayed by $\tau_{i}^{\left(k\right)}$, the amplification factor is set by the diagonal matrix ${\underline{\underline{A}}}^{\left(k\right)}$ and the mode-mixing unitary matrix ${\underline{\underline{V}}}^{\left(k\right)}$ is applied. Finally, the impulse response of the complete channel is given by:

(39) $${\underline{\underline{h}}}_{tot}\left(t\right)={\underline{\underline{h}}}^{\left(K_{amp}\right)}\left(t\right)*{\underline{\underline{h}}}^{\left(K_{amp}-1\right)}\left(t\right)*\cdots *{\underline{\underline{h}}}^{\left(1\right)}\left(t\right)*h_{CD}\left(t\right),$$

where $h_{CD}\left(t\right)=F^{-1}\left\{H_{CD}\left(\omega \right)\right\}$.

Note that, due to the random nature of ${\underline{g}}^{\left(k\right)}$ and ${\underline{\tau }}^{\left(k\right)}$ in each *k* span, we can generate an arbitrary number $N_{ch}$ of channel realizations of ${\underline{\underline{H}}}_{tot}\left(\omega \right)=F\left\{{\underline{\underline{h}}}_{tot}\left(t\right)\right\}$ for a given value of $\sigma_{g}$ and $\sigma_{gd}$.

Since we consider all the modes to be strongly coupled, the ${\underline{\underline{U}}}^{\left(k\right)}$ and ${\underline{\underline{V}}}^{\left(k\right)}$ matrices of each span *k* are modeled as unitary Gaussian random matrices obtained from a QR factorization of a complex random matrix whose elements have a zero mean and STD equal to 1. The two orthogonal matrices after QR factorization of two independent realizations of the random matrix are used as ${\underline{\underline{U}}}^{\left(k\right)}$ and ${\underline{\underline{V}}}^{\left(k\right)}$, respectively.

We consider a total number of $K_{amp}=100$ spans, each $\ell_{span}=50$ km long. For the fiber parameters, we used the multi-core fiber data reported in [41], considering the number of modes D=6 and the central wavelength $\lambda_{c}=1469$ nm. The selection of this multi-core fiber allow us to compare the results of this work with those presented in [28], and to obtain the fiber parameters needed for the numerical simulations from [41].

We take 2% as the underestimation dispersion factor that is applied to the dispersion coefficient $D_{CD}$ to obtain the residual CD experienced by the receiver. For the gain STD $\sigma_{g}$, we considered several values in the range of the systems referenced in [27]. For the numerical simulations, we compute a total of $N_{ch}$ = 10,000 realizations of the channel frequency response ${\underline{\underline{H}}}_{tot}\left(\omega \right)$ defined in (5).

### 5.2. Transmitter and Linear MIMO FSE Receiver Parameters

As described in Section 4, the transmitter uses a generalized PAM modulation and an square-root raised cosine for pulse shaping with a roll-off factor equal to α. We will show results of the numerical simulation for several values of α. The symbol period *T* has been set for a symbol rate $R_{s}=64$ GBaud. The FSE-based MIMO receiver has an oversampling factor $r_{ov}=2$, and a number of equalizer taps $N_{taps}=1000$ has been selected to ensure that it does not limit the receiver performance for the considered channel MDL.

### 5.3. Signal-to-Noise at the Input of the Receiver

The signal-to-noise ratio at the input of each mode ${\left(\frac{S}{N}\right)}_{in}\left(i\right)$ for i∈{0,…,D} is defined as:

(40) $${\left(\frac{S}{N}\right)}_{in}\left(i\right)=\frac{P_{in}\left(i\right)}{N_{0}/2}.$$

The signal-to-noise at the input of the receiver ${\bar{SNR}}_{in}$ in dB can be written as:

(41) $${\bar{SNR}}_{in}=10\cdot \log_{10}\left(\frac{\frac{1}{D}\cdot \sum_{i=1}^{D}P_{in}\left(i\right)}{N_{0}/2}\right)=10\cdot \log_{10}\left(\frac{1}{D}\cdot \sum_{i=1}^{D}{\left(\frac{S}{N}\right)}_{in}\left(i\right)\right),$$

and it is taken from the set of values in Table 1. $P_{in}\left(i\right)$ is the receiver input power in the mode *i* for the current channel realization.

### 5.4. Performance Loss Metric for FSE-Based MIMO Receiver

We define the performance loss metric (in dB) of the FSE-based MIMO receiver in MDL-impaired channels for certain mode *i* as:

(42) $$L\left(i\right)=SNR_{in}\left(i\right)-SNDR_{out}\left(i\right)$$

where $SNR_{\left(in\right)}\left(i\right)=10\cdot \log_{10}{\left(\frac{S}{N}\right)}_{in}\left(i\right)$, and ${\underline{SNDR}}_{out}=[SNDR_{out}\left(1\right),SNDR_{out}\left(2\right),\ldots ,$$SNDR_{out}{\left(D\right)]}^{T}$ is calculated as defined in [42] and Equation (28) in [43] for MIMO implementation of the FSE. Given a set of system model parameters, the numerical simulation will generate a total of $D\cdot N_{ch}$ values of L(i). The average loss AL is calculated for each channel realization of among the available $N_{ch}$ as:

(43) $$AL=\frac{1}{D}\sum_{i=1}^{D}L(i).$$

Two FSE-based MIMO receiver performance metrics can be extracted from the $D\cdot N_{ch}$ calculated values of L(i):

- $ML_{95}$ is defined as the 95th percentile of the L(i) distribution obtained for any optical channel realization and mode;
- $AML_{95}$ is defined as the 95th percentile of the AL distribution obtained for any optical channel realization.

The values for the parameters used in the simulation are summarized in Table 1.

### 5.5. Numerical Simulation Results

**Figure 6:**
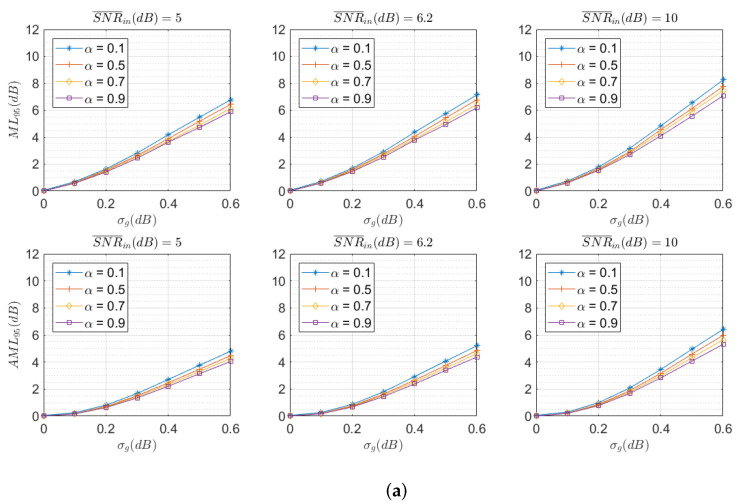

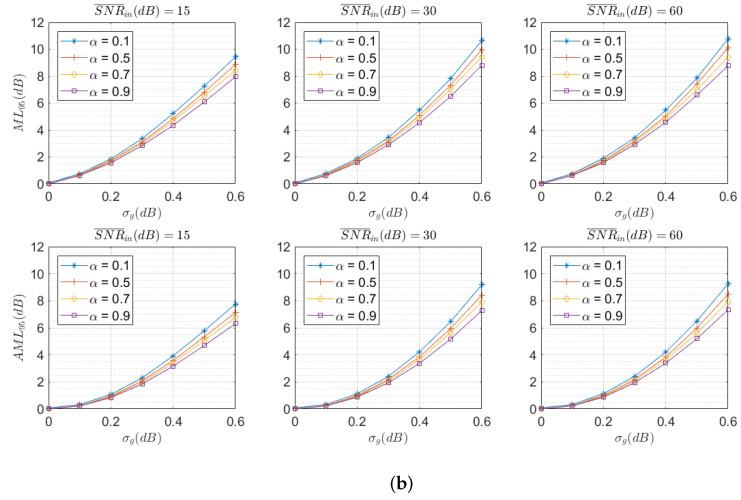
$ML_{95}$ (up) and $AML_{95}$ (down) as defined in Section 5.4 for ${\bar{SNR}}_{in}$ = 5, 6.2, and 10 dB (**a**) and ${\bar{SNR}}_{in}$ = 15, 30, and 60 dB (**b**) for different values of the transmitter roll-off factor α. Note that $\sigma_{g}=0$ corresponds to a channel without MDL.

The first result is focused on the impact of PAM pulses roll-off factor α and MDL level, represented by $\sigma_{g}$, on the SDM optical system performance. Figure 6 shows that for systems with transmitters using a higher α, the degradation is a bit lower. The effect is higher with increasing $\sigma_{g}$ for systems working at a high regime of ${\bar{SNR}}_{in}$, as seen in Figure 6b.

In practical systems, the allowable loss of ${\underline{SNDR}}_{out}$ in a channel with elements introducing MDL w.r.t. an ideal channel without MDL is around 1–2 dB. We can observe that, assuming a maximum degradation of 2 dB in the system with a 95% confidence, $\sigma_{g}$ values of the amplifiers should not exceed 0.2 dB. These results are in agreement with the capacity limits of a MIMO MMSE receiver and MDL channel calculated in [27].

**Figure 7:**
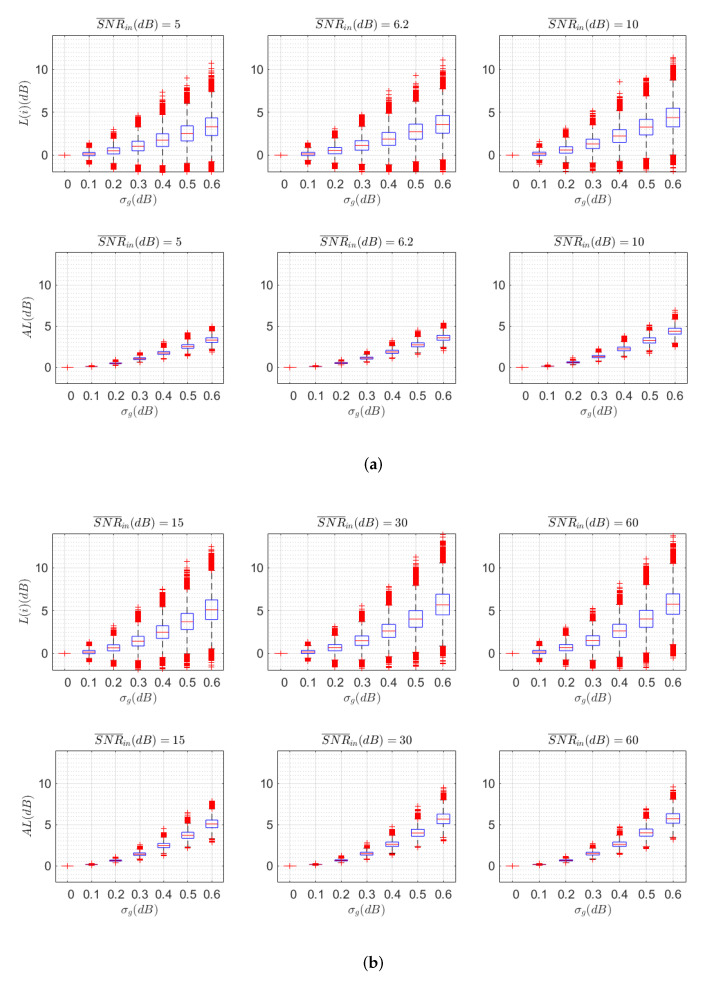
Probability distribution for AL as defined in (43) (up) and L(i) as defined in (42) (down) for ${\bar{SNR}}_{in}$ = 5, 6.2, and 10 dB (**a**) and ${\bar{SNR}}_{in}$ = 15, 30, and 60 dB (**b**). α = 0.9 for all graphs. Note that $\sigma_{g}=0$ corresponds to a channel without MDL.

A second result is presented in Figure 7, where the probability distribution of AL and ${\bar{SNR}}_{in}$ estimated from the analysis of all channel realizations is plotted. We are comparing different levels of SNR regimes, with ${\bar{SNR}}_{in}$ = 5, 6.2, and 10 dB (Figure 7a) and ${\bar{SNR}}_{in}$ = 15, 30, and 60 dB (Figure 7b), for a roll-off factor of α = 0.9. The upper and lower limits of the blue boxes represent the 25th and 75th percentiles respectively. The red line inside the box indicates the median of the metric. In case the distribution of values was Gaussian, the whisker bounds correspond to 2.7 times the STD of the metric or, in other words, the number of values between the upper and lower bounds of the whiskers contains 99.3% of the values. Values outside these limits are considered outliers and are individually represented by red crosses.

We make the following observations from Figure 7:

- The distribution of AL is not Gaussian, as we can observe by comparing the difference between upper and lower outliers for higher $\sigma_{g}$ values and their asymmetry;
- There are no negative values of AL, since the FSE MIMO receiver cannot improve on average the ${\bar{SNR}}_{in}$. However, by taking all values of L(i) for any received mode *i*, we can find that, for certain channels and modes, the FSE MIMO receiver can locally improve the $SNR_{in}\left(i\right)$ of a particular mode *i*, but always at the cost of another mode of the receiver;
- The performance degradation depends on the ${\bar{SNR}}_{in}$. In a low ${\bar{SNR}}_{in}$ regime (5 dB, Figure 7a), the degradation is measured lower in absolute values when compared to the high ${\bar{SNR}}_{in}$ regime (60 dB, Figure 7b);
- The STD of the performance degradation also depends on the ${\bar{SNR}}_{in}$. In the low ${\bar{SNR}}_{in}$ regime (5 dB, Figure 7a), the STD of the degradation is lower when compared to the high ${\bar{SNR}}_{in}$ regime (60 dB, Figure 7b);
- The performance degradation measured as AL is milder than measured as L(i) when more than 95% coverage of the channels is considered. Note that the difference is negligible when median values are taken into account.

## 6. Conclusions

This work explored long-haul fiber-optic SDM coherent systems with PAM raised-cosine pulses. We investigated the overall system performance under different configurations of the optical channel. For that purpose, we modeled this channel with a MIMO multi-span structure that included the several dispersion terms and modal losses factors.

It was demonstrated that the linear MMSE MIMO receiver completely eliminated ISI and crosstalk when the number of taps was sufficiently high and the optical channel was free of MDL. Moreover, the generic structure of the linear MMSE MIMO receiver could be simplified to a continuous-time matched filter and still retained the same properties. This observation paves the way for analog receivers that simply implement the matched filter of the optical channel, eliminating all channel impairments when MDL is negligible.

We also defined performance metrics to assess the losses of a linear MIMO receiver implemented using a MIMO FSE with an oversampling factor of two for an optical channel that exhibited significant MDL. We have shown that such loss depends on the transmitter PAM pulses roll-off factor and the SNR level at the receiver input. We also determined that the performance degradation could be limited by processing the *D* output modes together by averaging the *D* output SNDR values at the receiver.

This fact opens a way to exploit this loss compensation between modes at the receiver output. The design of specific forward error correction codes taking into account this aspect could improve the final performance of the system in terms of bit error rate. Constructing the message to be encoded, including bits or signals belonging to all modes, could improve system performance w.r.t. constructing messages with bits or signals from only one mode. 

## Figures and Tables

**Table 1:** Simulation parameters.

Parameter	Symbol	Value and Reference
Span length	$\ell_{span}$	50 km
Number of spans	$K_{amp}$	100
Number of spatial and polarization modes	*D*	6
Center wavelength	$\lambda_{c}$	1469 nm [41]
Modal dispersion	$\sigma_{\tau }/\sqrt{\ell_{span}}$	3.1 $\mathrm{ps}/\sqrt{\mathrm{km}}$ [41]
Dispersion coefficient	$D_{CD}=-\frac{2\pi c}{\lambda_{c}^{2}}\bar{\beta_{2}}$	20.1 ps/(nm·km) [41]
Underestimation dispersion factor	$U_{CD}$	2% [9]
Amplifier gain STD	$\sigma_{g}$	0, 0.1, 0.2, 0.3, 0.4, 0.5, 0.6 dB
Symbol rate	$R_{s}=1/T_{s}$	64 GBaud
Oversampling factor	$r_{ov}$	2
Roll off factor	α	0.1, 0.5, 0.7, 0.9
Number of channel realizations	$N_{ch}$	10000
Signal to noise ratio at the receiver input	${\bar{SNR}}_{in}$	60 30 15 10 6.2 5 dB [27]
Number of taps	$N_{taps}$	1000

## Data Availability

Not applicable.
